# Changes in Coagulation and Fibrinolytic Indices in Women with Polycystic Ovarian Syndrome Undergoing Controlled Ovarian Hyperstimulation

**DOI:** 10.1155/2014/731498

**Published:** 2014-10-13

**Authors:** Ying Huang, Yong Zhao, Ling Yan, Yun-Hai Chuai, Ling-Ling Liu, Yi Chen, Min Li, Ai-Ming Wang

**Affiliations:** ^1^Department of Reproductive Center, Navy General Hospital, Fucheng Road, Beijing 100048, China; ^2^Department of Obstetrics and Gynecology, Navy General Hospital, Fucheng Road, Beijing 100048, China

## Abstract

*Background*. Polycystic ovarian syndrome (PCOS) women undergoing in vitro fertilization and embryo transfer (IVF-ET) treatment always attain a low cumulative pregnancy rate disaccording with the satisfactory number of oocytes.* Objective*. We aim to evaluate the status of coagulation and fibrinolytic system in PCOS patients undergoing controlled ovarian hyperstimulation (COH) process.* Method*. Of the 97 women, 30 patients with PCOS composed the study group; 67 women of child-bearing age with normal endocrine function composed the control group. All participants underwent GnRH agonist standard long protocol, and plasma HCY, FVIII, FX, and D-dimer levels as well as hormone parameters were measured at day of full downregulation, hCG priming, and embryos transfer.* Results*. On day of full downregulation, FX levels were significantly higher in PCOS group (*P* < 0.01). On hCG priming day, FX and estrogen levels in PCOS group were higher than in the control group and FVIII levels were significantly lower on day of embryos transfer whereas FX and E2 levels were significantly higher in PCOS group.* Conclusion*. Hypercoagulable state during peri-implantation phase would probably lead to poor microcirculation of endometrium and be one of the most important disadvantages of successful implantation and subsequent clinical pregnancy.

## 1. Introduction

Polycystic ovarian syndrome (PCOS), the most common endocrine disease, affecting about 5–8% women of reproductive age [1], is defined by anovulation/oligoovulation, irregular menses, and androgen excess through 2003 Rotterdam criteria [2] and anovulatory infertility is the leading cause of infertility for these women.

Controlled ovarian hyperstimulation (COH), an important therapy for PCOS women after unsuccessful conventional ovulation induction, is the key component of IVF-ET and always obtains satisfactory number of oocytes with a low cumulative pregnancy rate [3]. During this approach, superphysiological doses of estrogen and progesterone were produced. Elevated levels of estrogen can change status of coagulation and fibrinolytic system and cause a hypercoagulable state which would probably lead to poor microcirculation of endometrium. To PCOS women, the number of oocytes is larger than in non-PCOS women; this change is more notable and would take poorer microcirculation of endometrium compared with non-PCOS women.

We aim to evaluate coagulation and fibrinolytic status during COH through detection of plasma HCY, FVIII, and FX as well as D-dimer, of which HCY is an endothelial injury marker, FVIII is a key procoagulant factor, FX plays an important part in the common pathway of intrinsic and extrinsic coagulation pathway, and D-dimer is the specific degradation products of fibrin indicating the formation of thrombosis. We ultimately aim to indirectly evaluate the status of endometrial microcirculation during periovulatory and peri-implantation period in PCOS patients undergoing COH process.

## 2. Materials and Methods

### 2.1. Patients

Ninety-seven women (22–38 years old) undergoing ovarian stimulation (recFSH, GnRH agonists in long protocol) from July 2012 until February 2013 at Reproductive Department of China Navy General Hospital were enrolled in this prospective study, of which thirty were PCOS diagnosed by 2003 Rotterdam criteria, which state that PCOS is diagnosed on the basis of having two out of three criteria: anovulation/oligoovulation, signs of clinical and/or biochemical hyperandrogenism, and polycystic ovaries on ultrasonography after exclusion of specific identifiable disorders (congenital adrenal hyperplasia, androgen-secreting tumors, Cushing's syndrome, thyroid dysfunction, and hyperprolactinemia) [2]. Body mass index (BMI) was calculated as body weight (kg) divided by height squared (m^2^). All of the patients signed an informed consent for their participation in the study. The fecundities of all male partners were normal according to World Health Organization criteria [4]. The same exclusion criteria as the PCOS group were used for the control group.

### 2.2. Controlled Ovarian Hyperstimulation

In PCOS patients, GnRHa (0.1 mg/day; Ferring, Kiel, Germany) was administered on the 21st day of spontaneous menstruation or a progestin-induced withdrawal bleed until hCG day. COH began on menstrual cycle day 2 after GnRHa administration with rFSH (Serono, Geneva, Switzerland). The starting dose of rFSH was 150 IU/day for all patients in both groups and this dose was subsequently adjusted depending on the ovarian response, as assessed by E2 levels combined with ultrasound. As soon as at least two leading follicles reached a mean diameter of ≥18 mm, 10000 IU of hCG (Pregnyl, Organon, The Netherlands) was administered and oocyte retrieval was performed by vaginal ultrasound 34–36 h after hCG administration.

### 2.3. Embryonic Grade, Embryo Transfer, and Luteal Support

On the third day after insemination, the morphology of each embryo was measured for number of cells, extent of fragmentation, and blastomere symmetry. Good quality embryos were defined as containing ≥6 equal-sized blastomeres with <20% fragmentation. Two or three embryos were transferred on day 3 after oocyte retrieval according to our routine procedure. In the luteal phase, all patients received intramuscular injection of 40 mg progesterone, starting from the day of oocyte retrieval until pregnancy testing 14 days after embryo transfer and dose increase or reduction depending on serum progesterone levels. Clinical pregnancy was defined as the presence of gestational sacs and fetal heart by ultrasound.

### 2.4. Blood Samples and Indices Measurements

Blood samples and measurements were undertaken after an overnight fast on day of pituitary downregulation amount to day 2 of the menstrual cycle after GnRHa administration (time 1), hCG injection (time 2), and 3 days after oocyte retrieval (time 3). Fasting venous blood was collected into fluoride oxalate tubes and sodium citrate tubes which were separated by cooled centrifugation at 3000 g for 10 min at 4°C to obtain serum and plasma separately. Serum and plasma samples were stored at −80°C until assayed.

Basal levels of FSH, LH, total T, and E2 were measured with the Siemens System (Siemens, Munich, Germany) in blood samples obtained from PCOS patients during a period of amenorrhea and from days 2–4 of normal menstrual cycle for normal women preceding IVF. Serum E2, P, LH, and FSH were measured by direct immunoassay on a Siemens analyser (Siemens, Munich, Germany).

Plasma HCY and D-dimer levels were determined by ELISA (enzyme linked immunosorbent assay), and FVIII activity was measured by the ability of a testing sample to correct clotting time of human FVIII-deficient plasma and reported as a percentage (George King Biomedical Inc.) [5]. FX activity was measured by percentage method and reported as a percentage.

### 2.5. Statistical Analysis

Statistical analysis was done with the SPSS 17.0. Clinical data of patients were statistically described as means ± SD. The serum hormone levels were analyzed using independent-samples *t*-test and *χ* ^2^ test was used for comparisons of the normal fertilization rates, implantation rates, and clinical pregnancy rates (see Table 3). In all analyses,* P* value < 0.05 was considered statistically different and* P* value < 0.01 indicated that difference was significant.

## 3. Results

There was no significant difference between the PCOS and control group for age, BMI, number of embryos transfers, endometrium thickness on transfer day, implantation rates, and clinical pregnancy rates (*P* > 0.05). Normal fertilization rates in the study group were significantly lower than in control group whereas basal testosterone, androstenedione, LH/FSH ratio, number of retrieved oocytes, and good quality embryos were significantly higher (see Table 1). There was no significant difference between the PCOS and control group for HCY, D-dimer, and progesterone levels during COH process. On day of pituitary downregulation, FX levels were significantly higher in the PCOS group (*P* < 0.01) and FX and estrogen levels in the PCOS group on hCG priming day were significantly higher compared with the control group. On day of embryos transfer, FVIII levels were significantly lower whereas FX and E2 levels were significantly higher in the PCOS group.

## 4. Discussion

The process of IVF involves using exogeneous hormones to achieve cycle control and stimulate the ovary and luteal support. During this process, supraphysiological estrogen levels can change status of coagulation and fibrinolytic system, which synthetically presents as a hypercoagulable state. Elevated levels of estrogen are a well-documented risk factor for thromboembolic complications which has been elaborated by Chan and Dixon [6]. From current available studies, it is clear that, with ovarian stimulation, both the coagulation and fibrinolytic systems are activated, which is closely associated with the supraphysiological dose of estrogen. However, whether these changes are sufficient by themselves to lead to unsuccessful implantation is yet unknown. As we all know, more estrogen is produced during ovarian stimulation process in PCOS women compared with normal women because of higher ovary responsiveness; thus we speculate that the coagulation and fibrinolytic systems probably are activated on a higher level in women with PCOS, which would likely bring about more adverse effects to successful implantation.

We performed this case-control study to evaluate differences of coagulation and fibrinolytic indices during IVF between women with PCOS in child-bearing age and the normal age-matched women without PCOS. We found that there was no significant difference in HCY levels between the two groups during the IVF process, and FVIII levels were significantly lower in PCOS group compared with the control group on day of embryos transfer amount to peri-implantation phase. FX levels in the PCOS group were significantly higher compared with the control group during the three periods, that is, day of pituitary downregulation, hCG priming, and embryos transfer. D-dimer levels, on day of embryos transfer, were higher than the control group though no statistic significance was reached. These findings indicate that coagulation and fibrinolytic systems were activated on a higher level in women with PCOS during COH process, and PCOS women reached a more hypercoagulable state compared with age-matched normal women (see Table 2).

Homocysteine (HCY) is an intermediate product formed by the breakdown of methionine and may translate into cysteine and cystathionine. High level of homocysteine which presents as hyperhomocysteinemia (HHCY) has been established in many PCOS patients [7] and it not only is an important factor for cardiovascular disease in PCOS women, but also is closely associated with vasculopathy and thrombogenesis [8]. Animal studies indicate that high level of homocysteine affects the blood vessel wall and causes a change in the endothelium and smooth muscle proliferation [8]. It may be a cause of changes and lesions in endothelial cells due to vascular fibrosis, which results in the activation of thrombogenesis, alterations in the coagulation system, and enhanced platelet activation [8]. It has also been postulated that high level of homocysteine may affect endometrium and contribute to the development of placental microvascular diseases, which adversely affect implantation process and maternal-fetal circulation [9], and all of these adverse effects may directly be associated with unsuccessful implantation and sustainable clinical pregnancy. Pacchiarotti et al. [10] reported that HHCY could cause microthrombi in the uterine vessels, compromise embryo implantation, and determine early pregnancy loss through coagulation dysfunction. It has been well elaborated that basal plasma homocysteine levels in PCOS patients are higher than in age-matched women [7] and high level of HCY is adverse to embryo implantation and early abortion [10]. Thus we suspected that plasma HCY levels were similarly higher in PCOS women compared with age-matched women undergoing COH process. However, in the present study, we did not find any difference in HCY levels between the two groups during the whole IVF process, and we analyzed possible reasons. In the present study, plasma HCY maintained at low level of 8.1 *μ*mol/L to 12.7 *μ*mol/L in both of the PCOS group and the control group, which was far lower than the standard of HHCY defined exceeding 17 *μ*mol/L [11] during IVF process. It was probably caused by pituitary downregulation effects which resulted in low HCY status and maintained a low level during the subsequent process. Besides, before and during IVF treatment, all participants received routinely supplement with folate which was one of the most important impact factors of plasma HCY level.

**Figure 1:**
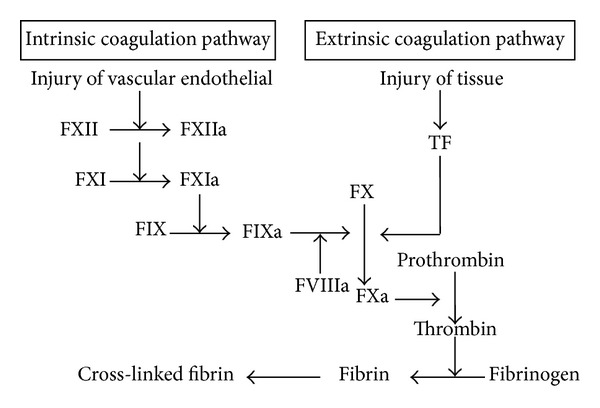
Coagulation pathway.

Coagulation factor VIII (FVIII) is an essential composition of the hemostatic mechanism, participating as a cofactor in the second burst of thrombin generation, which leads to clot formation (Figure 1). In response to injury, FVIII is activated and the active FVIII (FVIIIa) interacts with another coagulation factor called factor IX. This interaction sets off a chain of additional chemical reactions that form a blood clot. During normal menstrual cycle, plasma FVIII reached a peak level at luteal phase and was positively associated with high levels of estrogen [12]. Similarly, during IVF process, FVIII levels significantly elevated and were closely associated with elevated estrogen as described by Chan and Dixon [6] and Westerlund et al. [13]. However, there is no relevant study to compare FVIII levels between PCOS patients and the controls in former years. In our study, we found no significant difference in FVIII levels between the two groups on day of pituitary downregulation and hCG priming day. On day of embryos transfer, FVIII levels were significantly higher in PCOS group, which indicated a hypercoagulable tendency relative to the control group.

Coagulation factor X (FX) is a key enzyme which plays an important role in the common part of intrinsic and extrinsic coagulation pathway. FX is activated into FXa by factor IX (with its cofactor, factor VIII in a complex) and factor VII (with its cofactor, tissue factor in a complex) (Figure 1). FXa catalyses the conversion of prothrombin into thrombin and thus is the first member of the final common pathway or thrombin pathway [14]. In the present study, FX levels were significantly higher in the PCOS group on the three phases; those were day of pituitary downregulation, hCG priming, and embryos transfer. It indicated that there were more prothrombin converted into thrombin and suggested a higher level of procoagulant status in PCOS women than in non-PCOS women.

**Figure 2:**
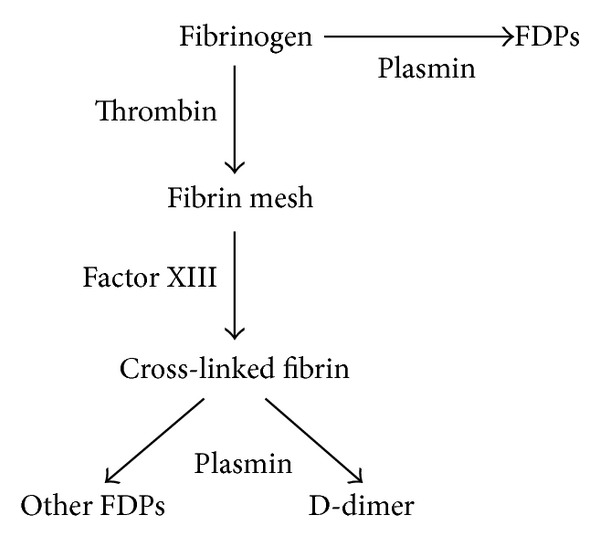
Fibrinolytic pathway.

D-dimer is the specific degradation products of cross-linked fibrin (Figure 2). D-dimers are not normally present in human blood plasma, except when the coagulation system has been activated, for instance, because of the presence of thrombosis or disseminated intravascular coagulation. Elevated level of D-dimer mainly indicates secondary hyperfibrinolysis which reflects elevated activation of coagulation system as well as the formation of thrombosis. In recent years, studies on relationship between estrogen and D-dimer focus on changes in D-dimer levels during normal menstrual cycle, and Giardina et al. [15] demonstrated a cyclic variation of D-dimer which was positively correlated with elevated estrogen. In the present study, we found that D-dimer levels in PCOS group, on day of embryos transfer (amount to peri-implantation phase), were higher than in the control group though no statistic significance was reached (*P* = 0.08). It is possible that higher levels of D-dimer observed in PCOS women was due to more intensive fibrinolytic activity secondary to hypercoagulation status in these women compared with the control group during IVF process. Since elevated D-dimer levels indicated thrombi formation, we speculate that there probably exists formation of microthrombi in the uterine vessels during peri-implantation period, which may probably lead to poor microcirculation of endometrium and do adverse effects to successful embryo implantation and maintenance of clinical pregnancy.

In the present study, we have not found any difference in implantation rates or clinical pregnancy rates between PCOS and the control group. However, clinical pregnancy rate of the PCOS group which is 20.0% compared with the control group 37.3% (*P* = 0.09) tend to be lower than the control group. In future study, more convinced results may be obtained by increasing the number of patients.

Certainly, plasma coagulation and fibrinolytic indices are not sufficient to evaluate status of microcirculation of endometrium, and thickness of endometrium on embryo transfer day is another important index which was not different between the two groups in the present study. In addition, observation of blood flow in endometrium on embryo transfer day via vaginal b-ultrasound is also an important method. In future study, it is sensible to evaluate blood flow of endometrium on embryo transfer day via vaginal b-ultrasound as well as detection of plasma coagulation and fibrinolytic indices to evaluate status of endometrium microcirculation in PCOS women during IVF process.

In the present study, on coagulation and fibrinolytic aspects, we compared HCY, FVIII, FX, and D-dimer levels during IVF process in PCOS and non-PCOS women. Through these indices, we indirectly evaluated the status of endometrium microcirculation during periovulation phase and peri-implantation phase in PCOS patients. In conclusion, during IVF process, there exists deeper hypercoagulable state and probably formation of microthrombus in the uterine vessels in PCOS women compared with non-PCOS women, which would probably lead to poor microcirculation of endometrium in PCOS women and influence successful embryo implantation as well as subsequent maintenance of clinical pregnancy. In addition, hypercoagulability on the success of embryo implantation after IVF may also determine the timing of preventing anticoagulant therapy in these women with history of early embryo loss.

## Figures and Tables

**(a):** 

Group	Age	BMI	Testosterone	Androstenedione	LH/FSH
	(y)	(kg/cm^2^)	(nmol/L)	(nmol/L)	
PCOS group	29.8 ± 2.80	22.48 ± 3.79	0.44 ± 0.16**	4.51 ± 1.72^∗∗^	1.07 ± 0.42**
Control group	30.15 ± 3.37	22.06 ± 2.83	0.31 ± 0.12	2.65 ± 1.02	0.60 ± 0.26

*P < 0.05, **P < 0.01.

**(b):** 

Group	FN	RON	GQE	ETN	En
PCOS group	19.9 ± 7.51**	15.57 ± 7.92**	6.93 ± 4.63*	2.03 ± 0.18	10.26 ± 1.14
Control group	14.67 ± 5.93	11.4 ± 5.07	5.28 ± 2.95	2.07 ± 0.27	10.08 ± 1.28

FN: number of follicles; RON: number of retrieved oocytes; GQE: good quality embryo.

ETN: number of embryos; En: endometrium thickness (mm).

*P < 0.05, **P < 0.01.

**(a):** 

Group	HCY	FVIII	FX
	(*μ*mol/L)	(%)	(%)
PCOS group			
Time 1	12.71 ± 15.95	58.48 ± 19.40	130.27 ± 19.51**
Time 2	8.05 ± 5.36	61.92 ± 21.52	117.41 ± 18.74^∗∗^
Time 3	8.11 ± 4.70	78.45 ± 19.87*	108.87 ± 15.16**
Control group			
Time 1	9.93 ± 5.39	52.98 ± 19.68	106.82 ± 18.90
Time 2	8.37 ± 5.39	55.04 ± 15.65	100.19 ± 20.07
Time 3	8.58 ± 6.04	67.51 ± 19.24	94.38 ± 18.39

*P < 0.05, **P < 0.01.

**(b):** 

Group	D-dimer	E2	*P*
	(mg/L)	(pmol/L)	(nmol/L)
PCOS group			
Time 1	0.18 ± 0.12	6.95 ± 4.57	0.90 ± 0.53
Time 2	0.15 ± 0.11	982.81 ± 520.49*	2.56 ± 1.72
Time 3	0.37 ± 0.27	525.62 ± 330.37**	306.1 ± 120.81
Control group			
Time 1	0.18 ± 0.11	9.40 ± 17.11	0.97 ± 0.50
Time 2	0.14 ± 0.08	777.10 ± 434.79	2.68 ± 1.28
Time 3	0.27 ± 0.18	341.31 ± 196.59	292.87 ± 110.48

*P < 0.05, **P < 0.01.

**Table 3:** Comparison of normal fertilization rates, implantation rates, and clinical pregnancy rates between the PCOS group and the control group [*n* (%)].

Indices	PCOS group	Control group	*X* ^2^ value	*P* value
Normal fertilization rates	349/467 (74.7)∗∗	634/764 (83.0)	12.27	<0.01
Implantation rates	11/30 (36.7)	26/67 (38.8)	0.04	0.841
Clinical pregnancy rates	6/30 (20.0)	25/67 (37.3)	2.86	0.09

*P < 0.05, **P < 0.01.
